# Full-body MR imaging: a retrospective study on a novel diagnostic approach for children sustaining high-energy trauma

**DOI:** 10.1007/s00068-021-01736-7

**Published:** 2021-07-19

**Authors:** Johanna Ludwig, Peter Heumann, Denis Gümbel, Ulrike Rechenberg, Leonie Goelz, Sven Mutze, Axel Ekkernkamp, Sinan Bakir

**Affiliations:** 1 Department of Trauma and Orthopaedic Surgery, BG Klinikum Unfallkrankenhaus Berlin gGmbH, Trauma Hospital Berlin, Warener Straße 7, 12683 Berlin, Germany; 2 Kellogg College, University Oxford, Oxford, UK; 3 Department of Trauma, Reconstructive Surgery and Rehabilitation Medicine, University Medicine Greifswald, Greifswald, Germany; 4 Department of Radiology and Neuroradiology, BG Klinikum Unfallkrankenhaus Berlin gGmbH, Berlin, Germany; 5 Institute for Diagnostic Radiology and Neuroradiology, University Medicine Greifswald, Greifswald, Germany

**Keywords:** Children, MRI, Trauma, Diagnostic

## Abstract

**Purpose:**

Severe accidents are the leading cause of long-term impairment and death in children. A common diagnostic procedure for children exposed to high-injury trauma is full-body contrast-enhanced CT (fbCT). However, the number of fbCT without detected injuries is relevant. In 2007, full-body MRI (fbMRI) was implemented as a diagnostic approach for children sustaining high-energy trauma.

The aim of this cross-sectional retrospective study was to analyze fbMRI as a diagnostic tool for children after high-energy trauma focusing on feasibility, radiological findings, and limitations.

**Methods:**

Diagnostics using fbMRI (from apex of the head to the pelvis) was performed if a child was stable and suffered a high-energy trauma in a Level I Trauma Center in Germany. 105 fbMRIs in patients exposed to high-energy trauma aged ≤ 16 years were performed between January 2007 and December 2018. Four fbMRIs were excluded as conducted for reasons other than trauma. Time between arrival in the emergency department and fbMRI, additional diagnostic procedures, injuries, and non-trauma related pathologies were analyzed.

**Results:**

Mean time between arrival in the emergency department and fbMRI was 71 min (± SD 132 min). Two scans were discontinued and changed to a faster diagnostic procedure. 45% of children had additional X-rays and 11% CT scans. The MRIs showed intracranial abnormalities in 27%, extremities injuries in 26%, spinal injuries in 18%, pelvic, and thoracic injuries in 7% of the cases.

**Conclusion:**

Overall fbMRI is a diagnostic alternative for hemodynamically stable, conscious children after high-energy trauma with the advantages of a radiation-free technique. However, MRI diagnostics take longer than CT scans. Prospective studies will be needed to identify the limiting factors of fbMRIs as primary diagnostic procedure compared to CT scans.

**Trial registration:**

German Clinical Trials Register (DRKS; DRKS00017015).

**Level of evidence:**

Case series, level of evidence V.

## Background

Multiple injuries following high-energy trauma are the leading cause of long-term impairment and death in children [1–3]. Optimal initial diagnosis and management of children who sustained high-energy trauma is of pivotal importance for providing treatment prerequisites that can help to reduce functional impairment and fatalities.

For adults, official guidelines outline a clear diagnostic path after high-energy trauma [4]. For children sustaining high-energy trauma, standard diagnostic pathways and management have rarely been evaluated and thus vary between trauma centers. There is broad consensus that ‘conventional’ CT scans of affected body regions, as determined by the trauma team, should be acquired for vitally threatened children requiring immediate intervention [5–13]. This management is highly dependent on the physical exam and primary survey by the trauma doctors, as their judgment decides upon further diagnostics.

Clinical studies have revealed that clinical examination in children may not be reliable and injuries are likely to be missed [5, 6, 14, 15]. However, using a full-body contrast-enhanced CT scan as a standard diagnostic tool poses great disadvantages and risks [16]. Children are more radiosensitive than adults. Radiation exposure during CT scans in childhood increases the risk for leukemia and brain tumors [17].

The risks of radiation exposure in the pediatric population have long been discussed. Studies have stressed that there is probably no threshold for carcinogenesis in children due to the extreme radiosensitivity of maturing tissue and organs. Also, the timespan for expressing the effects of ionizing radiation as cancer is much longer in children [18].

After accidents with severe trauma, radiation exposure from CT scans can amount to 18.8 (± 14.7) mSv [19]. Reviewed literature has calculated the risk for developing cancer after exposure with effective radiation doses of this magnitude to be elevated by about 1% [20]. Mortality increased by 0.07–0.18% depending on the scanned body region, the amount of radiation, and the age of the children [21, 22].

Nevertheless, most studies discussing radiation risks in children were published more than 10 years ago or are based on data obtained even before the millennium. The newer generation of CT scanners can perform low-dose CT protocols with reduced radiation exposure of 45–50% [23, 24]. Therefore, future studies should investigate the risks of radiation exposure in children after low-dose CT scans for reasonable comparisons with the benefits and disadvantages of whole-body MRI after high-energy trauma. The CT contrast medium poses the risk of allergic reactions and renal failure in susceptible patients.

Missed injuries, on the other hand, can lead to a devastating outcome [25]. Multiple studies exploring alternatives to CT imaging in the children population have been published, mostly focusing on examination protocols [6, 26, 27].

A new fbMRI protocol was introduced to the level I trauma center of the study site to examine children after having sustained high-energy trauma to address the dilemma between the disadvantages of CT imaging and the demand for precise and thorough initial diagnostics in a timely manner. Implemented in 2007 fbMRI provides an innovative diagnostic screening method without radiation exposure while possibly reaching a similar diagnostic sensitivity and specificity. FbMRI was first used in clinically stable patients having been exposed to high-energy trauma.

### Objectives

This retrospective study aimed to evaluate fbMRI as a diagnostic method for children exposed to high-energy trauma and to detect clinical limitations of this diagnostic approach.

## Methods

Diagnostics using fbMRI is considered by the trauma leader if a child:

- Suffered a fall from more than 3 m height.
- Was a passenger in a motorized vehicle during an accident with a velocity delta of more than 30 km/h or if other passengers were ejected or died from the same vehicle.
- Was affected as a pedestrian or cyclist in an accident.
- Cannot be sufficiently evaluated clinically.

During fbMRI, children were examined from the apex of the head to the pelvis (Table1). Naturally, both arms were part of the examination field during the relaxed supine position. A dedicated head coil and a body coil were used for the examinations. Table 1 gives the details of the examination protocol. The duration of the sequences amounts to 15–20 min. Three different MRI scanners were used during the observation period (Philips Panorama HFO 1 Tesla, Philips Intera 1.5 Tesla, Philips Achieva 3 Tesla; The Netherlands).

**Table 1:** MRI protocol for fBMRI in children

Body part	Sequences
Head	FLAIR sagittal
	TSE T2w axial
	FFE T2*w axial
Spine	STIR sagittal
	In cases of pathologies on STIR sequences: TSE T2w sagittal and axial, TSE T1w sagittal
Thorax	TSE T2w axial
Abdomen/pelvis	TSE T2w axial
	STIR coronal

An fbCT was to be performed in unconscious children with suspected severe brain injury and cardiovascularly affected children requiring stabilization prior to imaging.

This retrospective study was approved by the accountable ethics committee (University Medicine Greifswald, BB 016/19) and was registered with the German Clinical Trials Register (DRKS; DRKS00017015). No funding was received for this analysis.

Data generated or analyzed during the study are available from the corresponding author by request.

In this cross-sectional study, all patients aged 0–16 who underwent a fbMRI in our trauma level I hospital were identified through the picture archiving and communication system of the study site (Philips IntelliSpace Enterprise 4.4, Netherlands) from January 2007 to December 2018. MRI which were not conducted due to trauma or which were taken more than 24 h after trauma were excluded. Patients not receiving an fbMRI were not included in the study.

The analysis was conducted on retrospective data focusing on feasibility of the fbMRI according to the time of day, time between presentation to the emergency department to MRI, findings in the MRIs, and additionally required diagnostics measures.

### Patient and public involvement

Patients and the public were not involved in the design, conduction, reporting, or dissemination plans of this study.

### Statistical analysis

A statistical analysis was performed using SPSS software (IBM SPSS Statistics for Windows, Version 26.0. Armonk, NY: IBM Corp.). The mean values were compared for unpaired samples using Student’s *t* test with an alpha level of 0.05. Additionally, associations were tested by Pearson’s chi-squared test and Fisher’s exact test was used in the case of expected cell values less than *n* = 5. The analyzed MRIs had no missing data of the requested findings.

## Results

A total of 105 fbMRIs in patients aged ≤ 16 years were performed between January 2007 and December 2018. Four fbMRIs were conducted for reasons other than acute trauma. Overall, 101 met the inclusion criteria. Of these, two fbMRIs were discontinued, and diagnostics were changed to CT imaging as the faster imaging approach. Five fbMRIs had a limited diagnostic sensitivity and specificity due to motion artifacts.

40% of the children were female and 60% were male patients with an average age of 8.7 (± SD 3.2; range 2–16) years.

The most common causes for presentation were falls (36%), followed by accidents with a motorized vehicle as a pedestrian (33%), or as passenger in a car accident (12%) with a deceleration of more than 50 km per hour, another occupant ejected or dead. In 13%, fbMRI was performed after cycling accidents during which motorized vehicles were involved in 9%. Child abuse (2%) jumps off a tree meter springboard (1%), and sleigh accidents (1%) were less frequent events that lead to fbMRIs.

### Time to diagnosis

**Fig. 1:**
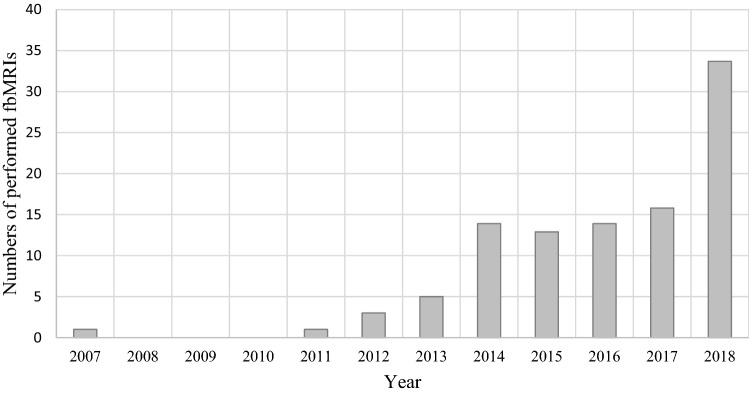
Use of fbMRI in children sustaining high-energy trauma during the observational period

fbMRI was first introduced in 2007. During the first 5 years, it was used scarcely as a diagnostic screening method. It has been increasingly applied since 2014 (Fig. 1).

**Fig. 2:**
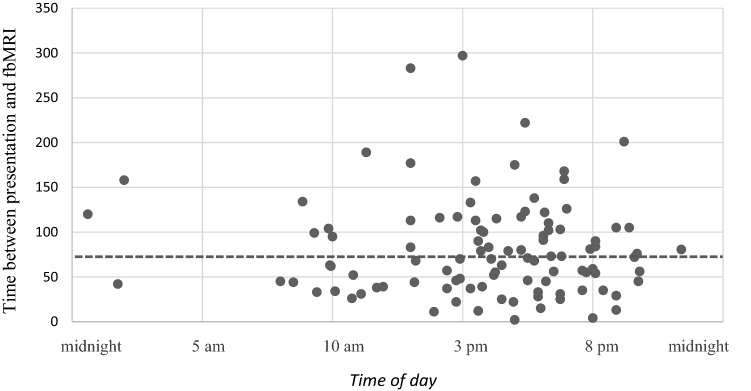
Distribution of time between presentation and MRI according to the time of day. The dotted line displays the average time of 71 min from presentation to fbMRI diagnostic

Average time from initial presentation to the emergency department (ED) to MR imaging was 71 min (range 2 min to 297 min). Most fbMRIs were performed between 10 am and 8 pm. Four fbMRIs were performed between 11 pm to 7 am (Fig. 2).

There was no significant difference between time to MRI in the timeframe between “7 am to 5 pm” and the timeframe “5 pm to midnight” (*p* = 0.169). The analysis showed a significant difference between “7 am to 5 pm”, and “midnight to 7 am” (*p* = 0.01) with a longer interval from presentation to MRI at nighttime.

The analysis according to the time needed to perform the fbMRI and diagnosed injuries in the MRI showed no significant differences between the time to fbMRI and the groups “injury/injuries” and “no injuries” (*t* test, sig. *R* = 0.731 *t* 0.345). Neither was there a difference between age of the child and the groups “injury/injuries” and “no injuries” (*t* test, sig. *R* = 0.540 t 0.614).

### Sedation and anaesthesia

Of all children undergoing fbMR imaging, 78% were not sedated. As the quality of MR imaging is highly depended on the patients’ compliance, 11% of all children had to undergo general anaesthesia before and only for imaging reasons to reduce motion artifacts. 8% of all children were sedated and ventilated at the trauma side, and 3% were sedated and ventilated in the emergency department due to their clinical presentation at arrival.

Of all children who underwent anaesthesia (22% of all children), 4% showed no injuries. 10% of the children who had to undergo anaesthesia to perform the MRI had no surgery, while 3% of the children who were intubated preclinically had no surgery. All children who underwent anaesthesia in the emergency department (ED) due to their clinical condition had to undergo surgery.

### Additional imaging

**Fig. 3:**
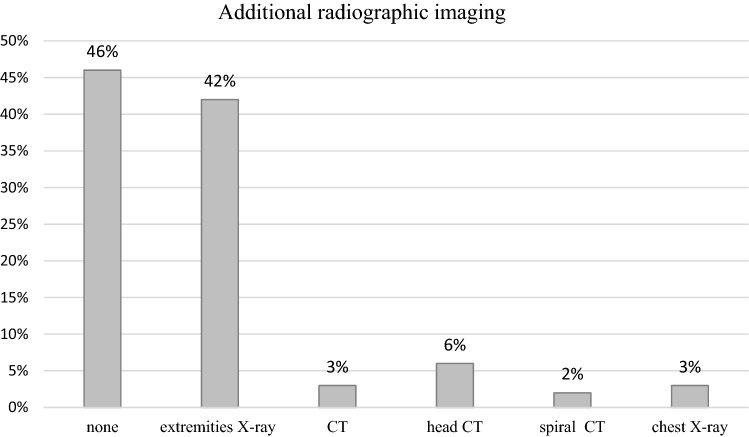
Diagnostic imaging performed in addition to fbMRI

More than half of the children (54%) received additional conventional radiographs (Fig. 3). 4% of the children received CT scans prior to MR imaging which were mainly head CTs and cervical spine CTs to rule out injuries that needed immediate intervention.

7% of the children had additional CT imaging after the fbMRI had been performed. Two whole-body contrast-enhanced CTs were performed after MR imaging was discontinued to change to the faster diagnostic method. In these cases, first, MRI sequences showed intracranial bleeding. Additional radiographs were performed if the fbMRI indicated a bone injury. All other suspected bone injuries and fractures were examined with conventional radiographs.

### Injuries

**Fig. 4:**
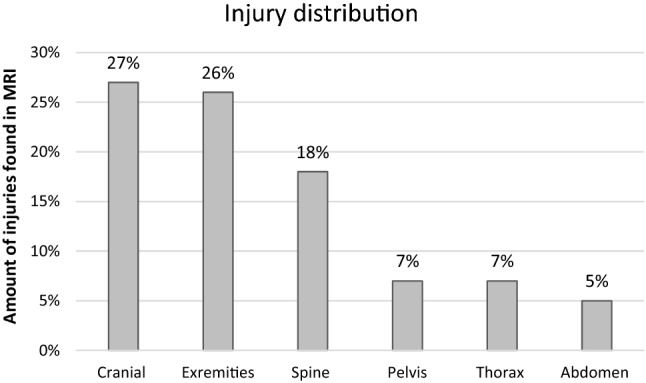
Distribution of injuries diagnosed in fbMRIs

Overall, 68% of the children examined showed injuries on fbMRI. 67% suffered multiple injuries. 6% accounted for minor injuries (i.e., bone bruises). Head MRI showed intracerebral injuries in 27% of the cases (Fig. 4).

These included haemosiderin stains (5%) and minor parenchymal bleeding (5%) without the need for operative therapy. Superficial hematomas were not further analyzed. In addition to injuries caused by trauma and additional pathological findings (Table 2), 25% of the MRIs showed low amounts of intra-abdominal fluid which was interpreted as physiological.

**Table 2:** Summary of MRI findings: injuries according to body parts and percentages in which they were found as well as non-trauma-related pathologies

Extremities		Spine and pelvis		Head		Thorax and abdomen		Additional findings	
Lower leg fracture	7%	Thoracic spine fractures	9%	Subarachnoid haemorrage	6%	Lung contusion	3%	Sinusitis	9%
Forearm fracture	5%	Pelvic fracture	7%	Skull fracture	5%	Soft tissue haematoma	2%	Lymphadeno-pathy	3%
Humerus fracture	4%	Bone bruise	6%	Haemosiderin stains	5%	Pleural effusion	2%	Arachnoid cyst	2%
Femur fracture	3%	Lumbal spine fracture	3%	Parenchyma hemorrhage	5%	Haematoma lesser pelvis	2%	Ovarian cyst	2%
Clavicle fracture	2%	Intraspinal haematoma	2%	Petrous bone fracture	3%	Liver injury	2%	Intraspinal synovial cyst	1%
Bone oedema	3%	Cervical spine fracture	1%	Epidural haematoma	1%	Bone bruise	1%	Ureteral enlargement	1%
Os metacarpale fracture	1%			Jaw fracture	1%	Retroperitoneal fluid	1%	Kidney cyst	1%
Foot fracture	1%			Nasal bone fracture	1%	Splen injury	1%	Mediastinal swelling	1%
								M. Perthes	1%
								Horseshoe kidney	1%
								Kidney malrotation or cyst	1%

## Discussion

Our data show that fbMRI can be used as a diagnostic screening tool for children sustaining high-energy trauma. Availability and time to fbMRI seem adequate in our institution during daytime for children in manageable stable conditions. Total radiation exposure during the diagnostic work-up with conventional radiographs and CT is reduced if whole-body CT scans are omitted. However, general anesthesia with its immanent risks and side effects was needed in one of ten patients to perform the fbMRI.

For adults whole-body contrast-enhanced CTs (wbCT) have become the standard diagnostic tool after exposition to high-energy trauma as it is widely accessible, non-invasive, quick, and precise [6]. In adults as well as in children, numbers of wbCT have increased in the last decades [28]. Children are more radiosensitive than adults [29]. Radiation exposure from CT scans in childhood increases the risk for leukemias and brain tumors [17], which has recently been confirmed by register-based studies [30].

 Sheppard et al. [31] showed that children who were exposed to one or more head CTs have an excess relative risk to develop brain tumors of 1.29 (95% confidence interval, 0.66–1.93).

Mueller et al*.* [32] conducted a prospective study to measure radiation exposure during wbCT scans after blunt trauma. In their study population, an average of three body parts were examined using CT scans in children. This led to a thyroid dose of 32.18 mGy (mean), increasing the risk for thyroid cancer to 71%, and a whole-body dose (mean 17.43 mSv) which aggravates the risk for tumors and leukemia.

The impact of radiation exposure led to the introduction of the “ALARA” principle. “ALARA” means “as low as reasonably achievable” and demands to decrease ionizing radiation exposure to a minimum [28, 33]. Sothi et al. have shown that preventing unnecessary CT scans can reduce scans in 8% of children in hospitals [34]. However, there is no doubt that if indicated the benefits of CT scans outweigh the risks [35].

In our study population, no anaesthesiological complication led to additional treatment or further diagnostics. However, due to the retrospective nature of the study, complications during anaesthesia cannot be ruled out entirely. Habre et al. [36] showed in a multi-center prospective study an incidence of perioperative severe critical events in 5.2% [36]. However, young age, medical history, and physical condition were shown to be the risk factors for serious critical events due to anaesthesia. Strøm et al. [37] analyzed the Danish data bank for pediatric anaesthesia (aged 2–17 years), showing serious adverse events in less than 0.3% of cases [37]. However, submitting 10% of the children to the risk of anaesthesia solely for the purpose of imaging needs to be opposed to the risks of radiation exposure through CT scans.

However, diagnostics of polytraumatized children or children that have been exposed to high-energy trauma remain a problem, because guidelines are not explicit and focus on the clinical exam (5–12). Clinical examination in children can be misleading [5, 6, 14, 15] which leads to a prevalence of non-diagnosed injuries of up to 9% [38].

Prospective studies show an incidence of 11–27% for missed fractures in children [14]. Heinrich et al*.* [39] screened children with a GCS of 12 or less 3 days after trauma using a triple-phase technetium radionucleotide bone scan screening evaluation. They showed that the false-negative rate of plane radiographs was up to 2%.

Missed or delayed diagnosis of fractures in children can have serious consequences and should be avoided to reduce complications and morbidity [39].

MR imaging can detect occult fractures that were missed on X-rays [40, 41]. Our study does not provide information on possibly missed fractures on plane radiograph due to its retrospective design. The fbMRI provides a screening method to precisely conduct plane radiographs afterwards. However, plane X-rays were still conducted in a high number of children. Furthermore, CT scans were performed in one of ten children.

While our study used a full-body MRI, other studies have investigated MRI for specific body parts in children. Cohen et al. stated “Rapid protocols for head MRI after acute brain injury in pediatric patients have been shown to reduce examination times while maintaining a high level of accuracy. They require only 3–6 min inside the scanner and ultimately a minimal level of sedation in agitated children” [42, 43]. Using MRI for diagnostics in children therefore seems to be increasing.

Head injuries are the major cause of mortality in children [44]. The concern to miss head injuries in pediatric patients has led to an increase in head CT scans [7, 28]. In Canada, CT scans of children after head injuries have increased from 15% in 1995 to 53% in 2005 [7].

One in ten children showed intracranial haemosiderin stains or small parenchyma hemorrhage, both of which are diagnosed on MR imaging more sensitively than on CT [45, 46]. Whether these findings are of clinical relevance or even lead to long-term impairment in these children has not yet been evaluated.

There are limitations of this study worth mentioning. First, as this is a new diagnostic approach no prediction about sensitivity and specificity of the fbMRI has been investigated.

Second, due to its retrospective nature, fbMRI could not be compared directly to the diagnostic performance of thorough clinical examination and wbCT. Therefore, feasibility and findings are not compared to the diagnostic standard. Thirdly, the final decision to perform an fbMRI depended on the trauma doctor in charge. Finally, this study does not imply cost analysis of additional costs due to fbMR imaging or an analysis of a possible additional risk due to required anesthesia.

To date, reliable data of MRI use in children in emergency situations are low [28]. The available studies focus on children presenting to an emergency department for multiple reasons not on trauma in particular. The use of MRI in these studies is limited to body-part specific MRIs [47–49]. This study introduced fbMRIs as a screening method in children sustaining high-energy trauma.

So far, these retrospective study results allow only for the cautious recommendation to consider performing fbMRI in selected children after high-energy trauma to rule out relevant injuries in unpredictable cases and to avoid missing discreet injuries. Further prospective results are still needed to assess these additional diagnosis's clinical and legal impact and propose universal guidelines for acute imaging after high-energy trauma in children.

## Data Availability

No study data or additional data are available.
